# Aberrant Energy Metabolism in Tumors and Potential Therapeutic Targets

**DOI:** 10.1002/gcc.70008

**Published:** 2024-11-25

**Authors:** Shuhao Fan, Jianhua Guo, Hui Nie, Huabao Xiong, Yong Xia

**Affiliations:** ^1^ Shandong First Medical University Jinan Shandong People's Republic of China; ^2^ College of Medical Engineering Jining Medical University Jining Shandong People's Republic of China; ^3^ Institute of Immunology and Molecular Medicine, Jining Medical University Jining Shandong People's Republic of China

**Keywords:** energy metabolism abnormality, glucose metabolism, glutamine metabolism, lipid metabolism, oxidative phosphorylation

## Abstract

Energy metabolic reprogramming is frequently observed during tumor progression as tumor cells necessitate adequate energy production for rapid proliferation. Although current medical research shows promising prospects in studying the characteristics of tumor energy metabolism and developing anti‐tumor drugs targeting energy metabolism, there is a lack of systematic compendiums and comprehensive reviews in this field. The objective of this study is to conduct a systematic review on the characteristics of tumor cells' energy metabolism, with a specific focus on comparing abnormalities between tumor and normal cells, as well as summarizing potential targets for tumor therapy. Additionally, this review also elucidates the aberrant mechanisms underlying four major energy metabolic pathways (glucose, lipid, glutamine, and mitochondria‐dependent) during carcinogenesis and tumor progression. Through the utilization of graphical representations, we have identified anomalies in crucial energy metabolism pathways, encompassing transporter proteins (glucose transporter, CD36, and ASCT2), signaling molecules (Ras, AMPK, and PTEN), as well as transcription factors (Myc, HIF‐1α, CREB‐1, and p53). The key molecules responsible for aberrant energy metabolism in tumors may serve as potential targets for cancer therapy. Therefore, this review provides an overview of the distinct energy‐generating pathways within tumor cells, laying the groundwork for developing innovative strategies for precise cancer treatment.

**Abbreviations**
6PGDH: 6‐phosphaogluconate dehydrogenase
6PGL: 6‐phosphogluconolactonase
ACACA: acetyl‐CoA carboxylase alpha
ACC: acetyl‐CoA carboxylase
acetyl‐CoA: acetyl coenzyme A
ACLY: ATP‐citrate lyase
ACSS: acetyl coenzyme A synthase
ADP: adenosine diphosphate
ALYREF: Aly/REF export factor
AMPK: adenosine monophosphate‐activated protein kinase
AR: androgen receptor
ASCT2: alanine‐serine‐cysteine transporter 2
ATP: adenosine triphosphate
ATP5B: ATP synthase β‐subunit
BCA: breast carcinoma
CAT1/CCAT: carnitine palmitoyltransferase I
CCA: cholangiocarcinoma
CD36: cluster of differentiation 36
ChIP‐seq: chromosome immunoprecipitation‐sequencing
Complex I: NADH; ubiquinone oxidoreductase
Complex II/SDH: succinate dehydrogenase
Complex III: Cytochrome bc1
Complex IV/COX: Cytochrome c oxidase
Complex V: ATP synthase
CREB: cyclic‐AMP response element‐binding protein
CREB‐1: cyclic‐AMP response element‐binding protein 1
ERK: Ras/extracellular signal‐regulated kinase
ETCs: electron transport chains
F‐1,6‐P: fructose‐1,6bisphosphate
F‐2, 6‐BP: fructose 2,6‐bisphosphate
F‐6‐P: fructose‐6phosphate
FA: fatty acids
FADH2: flavin adenine dinucleotide
FAO: FA oxidation
FASN: fatty acid synthase
FH: fumarate hydratase
FoxO1: Forkhead box protein O1
G‐6‐P: glucose‐6‐phosphate
G6PDH: glucose‐6‐phosphate dehydrogenase
GLS: glutaminase
GLS1: renal glutaminase
GLS2: hepatic glutaminase
GLUD: glutamate dehydrogenase
GLUTs: glucose transporters
HCC: hepatocellular carcinoma
HIF‐1α: hypoxia‐inducible factor‐1 alpha
HK: Hexokinase
HMGCR: 3‐hydroxy‐3‐methylglutaryl‐CoA reductase
HNSC: head and neck squamous cell carcinoma
IDH: isocitrate dehydrogenase
IKKβ: inhibitor of nuclear factor kappa‐B kinase subunit beta
LDHA: lactate dehydrogenase A
LDs: ipid droplets
lncRNA: long non‐coding RNA
MAPK: mitogen‐activated protein kinase
MIEF2: mitochondrial elongation factor 2
mRNA: messenger RNA
mtDNA: mitochondrial DNA
mTOR: mammalian target of rapamycin
NADH: nicotinamide adenine dinucleotide
NDUFA4L2: NADH dehydrogenase 1 alpha subcomplex, 4‐like 2
NF‐κB: nuclear factor kappa B
NRF2: nuclear factor erythroid 2‐related factor 2
NSUN3: NOP2/Sun RNA methyltransferase 3.
OSCC: oral squamous cell carcinoma
OXPHOS: oxidative phosphorylation
P2X7: a kind of ATP receptor
PDK2: pyruvate dehydrogenase kinase 2
PFK‐1: phosphofructokinase‐1
PGC1α: PPARγ co‐activator 1α
PGM: phosphoglycerate mutase
PHB: prohibitin
PI3K: phosphoinositide 3‐kinase
PK: Pyruvate kinase
PKM1: PK muscle isoenzymes M1
PKM2: PK muscle isoenzymes M2
PPARγ: peroxisome proliferator‐activated receptor‐γ
PPP: pentose phosphate pathway
PTEN: phosphatase and tensin homolog deleted on chromosome 10
PUFA: polyunsaturated fatty acid
QCR2: ubiquinol‐cytochrome C reductase core protein II
ROS: reactive oxygen species
SCD: stearoyl‐CoA desaturase
SCO2: cytochrome c oxidase assembly protein 2
SDH: succinate dehydrogenase
SLC2A3: solute carrier family 2 member 3
SN2: a kind of amino acid transporter proteins
STAT3: signal transducer and activator of transcription 3
TAG: triglycerides
TCA: tricarboxylic acid
Tcl1: T‐cell leukemia 1
TKT: transketolase
TME: tumor microenvironment
UQCRB: ubiquinol‐cytochrome C reductase‐binding protein
USP22: ubiquitin‐specific protease 22
USP30: ubiquitin‐specific peptidase 30
VEGF: vascular endothelial growth factor
α‐KG: α‐ketoglutarate

## 1 Introduction

Cancer is one of the most significant diseases that affect human longevity, and energy metabolism has recently emerged as a central focus in oncology research. One characteristic of living organisms is their metabolism, which can be categorized into energy and substance metabolisms. Energy metabolism involves adenosine triphosphate (ATP) hydrolysis and synthesis pathways, which depend on the intracellular metabolism of substances accompanied by nicotinamide adenine dinucleotide (NADH) renewal [1]. This process serves as the primary energy source for life activities, requiring energy for macromolecule synthesis, transmembrane transport, and mechanical function (contraction and cell activity) of macromolecules at the molecular level in normal cells. These essential activities are achieved via reactions that involve ATP synthesis and hydrolysis. Furthermore, tumor cells' apparent growth, multiplication, migration, invasion, and angiogenesis are high‐energy‐consuming processes. The energy metabolism‐related pathways include glucose metabolism (glycolysis and pentose phosphate pathway [PPP]), glutamine metabolism, lipid metabolism, mitochondrial activity (the tricarboxylic acid [TCA] cycle and oxidative phosphorylation [OXPHOS]), and autophagy. Tumor cells exhibit metabolic characteristics that differ from those of normal cells to meet complex energy requirements because of their unlimited potential for proliferation and abnormal cellular energy [2].

How do cancer cells undergo metabolic reprogramming to adapt to diverse environments and develop altered metabolic pathways, thereby acquiring resistance against external environmental stressors? The heterogeneity of tumor metabolic reprogramming refers to the distinct metabolic characteristics exhibited by various tumor cell types and homogeneous tumor cells during different progression stages [2, 3]. These metabolic characteristics are closely associated with the complex tumor microenvironment (TME), comprising tumor cells, immune cells, cellular signaling factors, fibroblasts, and extracellular matrix. Furthermore, the most prominent characteristic of tumor cells' energy metabolism is their tendency to use the glycolytic pathway to produce ATP and metabolic intermediate, sustaining tumorigenesis through aerobic glycolysis, even if in the presence of sufficient oxygen, rather than relying on mitochondrial OXPHOS. This phenomenon is known as the Warburg effect [4]. Although studies involving the characteristics of tumor energy metabolism and the development of antitumor drugs that target energy metabolism are currently popular topics, systematic compendiums and comprehensive reviews in this direction are limited. Thus, this review presents a comprehensive assessment of recent studies on molecules that regulate complex energy metabolism networks and evaluates their potential as therapeutic targets for cancer treatment, thereby providing theoretical insights into future research in this field.

## 2 Reprogramming of Glucose Metabolism in Tumor Cells

**FIGURE 1:**
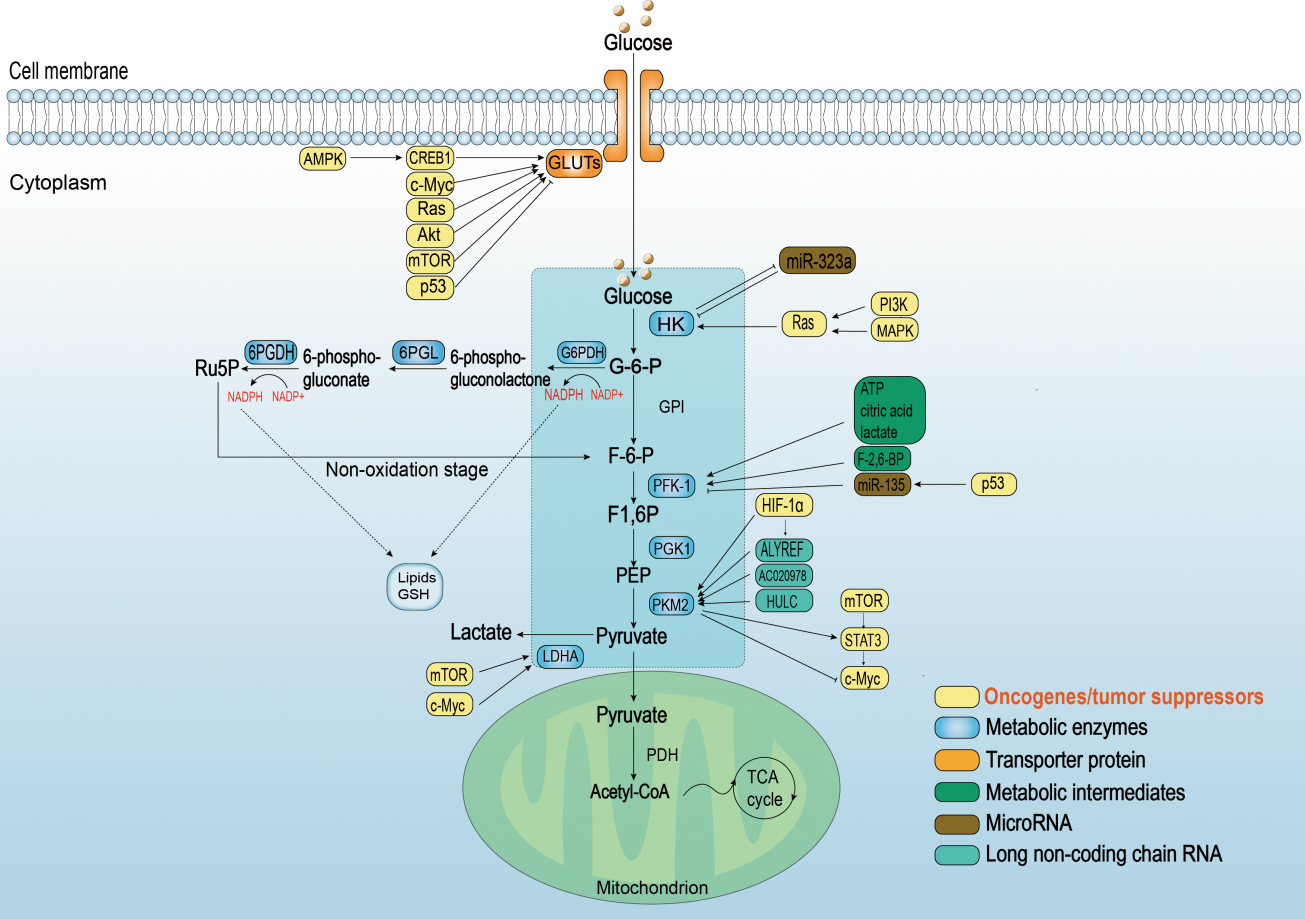
Regulation of glucose metabolism in cancer cells. Glucose metabolism includes glycolysis and the pentose phosphate pathway (PPP) in the cytoplasm, and the TCA cycle in the mitochondria. In tumor cells, most of the enzymes and glucose transport proteins of the glucose metabolic pathway are altered in expression, and the regulatory mechanisms of these alterations are partially represented in the figure, as detailed in the original document. 6PGDH, 6‐phosphogluconate dehydrogenase; 6PGL, 6‐phosphogluconolactone; CA, citric acid; F1,6P, fructose‐1,6bisphosphate; F6P, fructose‐6phosphate; G6P, glucose‐6phosphate; G6PDH, glucose6‐phosphate dehydrogenase; GLUTs, glucose transporters; GPI, glucose‐6‐phosphateisomerase; HK, hexokinase; LDHA, lactate dehydrogenase A; PDH, pyruvate dehydrogenase; PFK‐1, phosphofructokinase‐1; PGK1, phosphoglycerate kinase 1; PKM2, pyruvate kinase isozyme type 2; Ru5P, ribulose 5‐phosphate; TCA, cycle tricarboxylic acid cycle.

Cells primarily obtain energy from the pathways of glucose metabolism in the body, which include the TCA cycle, glycolysis, and some branch pathways such as the PPP. Oncogenic mutations can promote nutrient uptake, particularly glucose, beyond the energy requirements for cell proliferation. Additionally, the Warburg effect indicates that cancer cells stably generate energy through the aerobic glycolytic pathway, even when oxygen is sufficient to support OXPHOS [4]. Why do tumor cells with higher proliferation rates choose a less efficient productivity approach? They opt for this approach to compensate for the declining functional level of mitochondrial productivity and the following reasons: (1) To ensure the structural and functional integrity of passaged daughter cells. Proliferating cells must undergo a complete replication of their contents, which requires a large number of nucleic acids, amino acids, and lipids, among others. The glycolytic process generates ample biomass synthesis precursors during ATP production to sustain the biosynthetic building block requirements for cell proliferation, whereas OXPHOS converts carbon from glucose into CO_2_, which cannot be used to synthesize biomolecules during tumorigenesis. Specifically, the abundance of resources is prioritized over the efficiency of ATP production [5, 6]. (2) Energy production from aerobic glycolysis is inefficient; however, the rate is significantly higher than that of OXPHOS, and the amount of ATP produced per unit of time is even higher than that of OXPHOS under the same circumstances, which considerably aligns with rapid tumor proliferation requirement [5]. (3) Even slight perturbations in the ratio of ATP/adenosine diphosphate (ADP) can decline cell growth. Inefficient ATP production results in less interconversion of ADP and ATP, which is beneficial for maintaining ATP/ADP stability and promoting cell growth and proliferation [6]. Therefore, tumor cells gain owna competitive advantage precisely because of the numerous abnormal activations in these glucose metabolism‐signaling pathways (shown in Figure 1).

### 2.1 Dysregulation of Glycolytic Pathways and Key Enzymes in Tumorigenesis

High glycolysis level, an extraordinary abnormality of energy metabolism in cancer, is a pivotal cause of cachexia in various cancers and hinders the progression of the antitumor immune response [7, 8]. Therefore, transporter proteins, key enzymes, and transcription factors in the glycolytic pathway have emerged as potential targets for intervention in tumor therapy research. Which key enzymes in the glycolytic pathway play a critical role in tumorigenesis?

Glucose transporters (GLUTs)‐mediated glucose uptake crossing the plasma membrane are the first step and an important rate‐limiting step in glycolysis. The GLUT protein family has 14 members (GLUT1–14) that facilitate energy optimization and competitive advantage in diverse cancer species, depending on their different hexose affinities. GLUTs are upregulated in most tumors, with almost all tumor cells overexpressing GLUT1, assuming a glucose transportation function, while some tumor tissues overexpress other isoforms absent in normal cells [9]. GLUT1 expression is upregulated by the oncogenes, c‐Myc, Ras, Atk, and mammalian target of rapamycin (mTOR) but repressed by the tumor suppressor gene p53 [10]. Although GLUT1 overexpression is universal, GLUT3, encoded by solute carrier family 2 member 3 (SLC2A3), has a higher affinity for glucose and mediates glucose utilization, preferentially fueling nucleotide synthesis. In breast cancer brain metastases, cyclic‐AMP response element‐binding protein (CREB), an important transcription factor in brain cancer development, positively regulates GLUT3 expression [11]. Energy stress or glucose deficiency in the TME activates adenosine monophosphate‐activated protein kinase (AMPK), which activates CREB1 (a proto‐oncogene) to induce GLUT3 expression in colorectal cancer cells by anchoring the CREB1 binding site on the SLC2A3 promoter. However, the effect of GLUT3 on tumor cell growth was significantly higher than that of GLUT1 in this particular context [12].

Hexokinase (HK) is an initial rate‐limiting enzyme in glucose metabolism, catalyzing ATP‐dependent phosphorylation of glucose to generate glucose‐6‐phosphate (G‐6‐P), thereby modulating the magnitude of glucose flux. Depending on the encoding gene, HK, which is also known as glucokinase, exists in mammals in four isoforms as follows: HK1, HK2, HK3, and HK4. Cancer cells express high HK2 levels, distinguishing them from normal cells. Studies have shown that HK2 expression in cholangiocarcinoma, head and neck squamous cell carcinoma (HNSC), lung squamous cell carcinoma, prostate adenocarcinoma, stomach adenocarcinoma, esophageal carcinoma, glioblastoma multiforme, bladder urothelial carcinoma, and other tumors is significantly higher than that in the corresponding normal tissues [13]. Oncogenic Ras has been shown to mediate HK2 overexpression (both phosphoinositide 3‐kinase [PI3K] and mitogen‐activated protein kinase [MAPK] signaling contribute to this phenomenon), which was significantly elevated in individual tumors isolated from KRas‐induced mouse lung tumors, without significant change in HK1 protein levels [14]. MicroRNA‐323a (MiR‐323a) negatively regulates tumor cell growth and progression as a tumor suppressor in various tumor cells [15, 16, 17]. It is expressed at low levels in pancreatic cancer cells and is enriched in glucose metabolism pathways to negatively regulate the downstream factor HK2, resulting in increased glycolytic flux. Moreover, HK‐2 cell overexpression also eliminated the effect of miR‐323a overexpression [18].

Phosphofructokinase‐1 (PFK‐1) is the second rate‐limiting enzyme in glycolysis that utilizes ATP to catalyze fructose‐6‐phosphate (F‐6‐P) conversion to fructose 1,6‐diphosphate (F‐1, 6‐BP). Depending on the energy metabolism requirements of different mammalian tissues, the three isomers of PFK‐1, which are liver‐, muscle‐, and platelet‐type PFK, exist in different composition ratios. Studies have shown that PFK‐1 expression is significantly higher in cervical carcinoma cases than in controls and is overexpressed in breast carcinoma (BCA) [19, 20]. PFK‐1 downstream products, including ATP, citric acid, and lactic acid, induce the dissociation of the fully activated tetrameric form of PFK‐1 into the minimally active dimeric form. This phenomenon creates a negative feedback mechanism that reduces glycolytic flux [21]. However, fructose 2,6‐bisphosphate (F‐2, 6‐BP), which is the product of F‐6‐P catalyzed by 6‐phosphofructo‐2‐kinase/fructose‐2,6‐biphosphatase 3, is considered the most potent allosteric activator of PFK‐1 and can effectively counteract the negative feedback mechanisms of PFK‐1 downstream products [22]. Moreover, glutamine deprivation directly targeted the repression of PFK‐1 expression during glycolysis in pancreatic ductal adenocarcinoma cells via mutant p53‐induced miR‐135, which links glycolysis and glutamine metabolism in tumor cells [23].

Pyruvate kinase (PK) is the final rate‐limiting enzyme in glycolysis regulation that catalyzes ATP and pyruvate production from phosphopyruvate. Four different isoforms, which are liver‐type PK, erythrocyte‐type PK, and PK muscle isoenzymes M1 and M2 (PKM1 and PKM2), were expressed using different promoters and alternative splicing of the PKM gene [24]. Previous studies have mainly discussed PKM2, due to its high expression in tumor cells and its association with poor prognosis [21, 24]. Additionally, studies on bladder cancer showed that the Aly/REF export factor (ALYREF) stabilized PKM2 expression by binding to the 3′‐untranslated region 5‐methylcytidine site in the messenger RNA (mRNA) of PKM2. Meanwhile, hypoxia‐inducible factor‐1 alpha (HIF‐1α) can directly activate PKM2 transcription and indirectly upregulate its expression ALYREF‐dependently to produce tumor‐promoting effects. The discovery of the HIF‐1α/ALYREF/PKM2 signaling pathway provides another novel potential therapeutic target for the BCA treatment [25]. PKM2 is also a target of many long noncoding RNA (lncRNA); for example, the lncRNA highly upregulated in liver cancer (HULC) significantly increases PKM2 expression in hepatocellular carcinoma (HCC) by enhancing cyclin D1 through the autophagy‐miR675‐PKM2 signaling pathway, supporting the oncogenic role of HULC in HCC stem cells [26]. The lncRNA AC020978 is upregulated, as a response to glucose starvation and lack of oxygen, and HIF‐1α directly induces its expression. Mechanistic investigations demonstrated that AC020978 directly interacted with PKM2, enhancing PKM2 protein stability. Additionally, AC020978 promoted the nuclear translocation of PKM2 and augmented its transcriptional activity [27]. Recent studies have shown that PKM2 knockdown does not prohibit bladder cancer development but impairs HRas‐driven tumor growth and maintenance by suppressing vascular endothelial growth factor (VEGF)‐related angiogenesis by reducing PKM2 and signal transducer and activator of transcription 3 (STAT3) complex formation and nuclear translocation. The PKM2–STAT3–HIF1a/VEGF signaling axis may play a key role in bladder cancer and serve as a feasible therapeutic target [28]. However, a gastric cancer study identified connections between mTOR/PKM2 and STAT3/c‐Myc signaling pathways, constituting a molecular network that jointly regulates the energy metabolism in gastric cancer [29]. The abovementioned study showed that mTOR, STAT3, and c‐Myc are present on one axis from upstream to downstream and that PKM2 is located upstream of both STAT3 and c‐Myc to play positive and negative regulatory roles, respectively. Thus, the co‐inhibition of PKM2 and c‐Myc is a potential therapeutic strategy for gastric cancer and is superior to single inhibition [29]. PKM2 has gained recognition as a significant target in cancer therapy [24].

### 2.2 Disorder of Pentose Phosphate Pathway and the Key Molecules

PPP deviates from glycolysis, which is a pivotal step in glucose metabolism. It is required for ribonucleotide synthesis and is the main source of nicotinamide adenine dinucleotide phosphate (NADPH) for synthesizing various substances, consequently providing reduced glutathione as the major scavenger of reactive oxygen species (ROS) to protect the cells. Particularly, tumor cells with high‐level ROS that can accelerate metabolism and are more sensitive to oxidative stress‐induced cell damage. However, these intermediates can provide raw materials, such as 5‐P‐ribose, nucleotides, and 4‐P‐erythritose, for synthesizing other substances [30]. PPP is classified into two main processes as follows: oxidative and non‐oxidative stages; the rate‐limiting enzymes in the oxidative stage are discussed below. The oxidative stage that produces NADPH and ribonucleotides, includes three irreversible reactions and is the main rate‐limiting phase of the PPP with the following three key rate‐limiting enzymes: glucose‐6‐phosphate dehydrogenase (G6PDH), 6‐phosphogluconolactonase (6PGL), and 6‐phosphaogluconate dehydrogenase (6PGDH). Conversely, the non‐oxidative branch comprises a series of reversible reactions that produce additional intermediates, such as F‐6‐P and glyceraldehyde‐3‐phosphate, which re‐enter the glycolytic pathway and can be converted into pentose phosphate [30]. Many tumor lesions promote glucose influx into the PPP pathway, and are regulated by various oncogenes such as PI3K, mTOR complex 1 (mTORC1), K‐ras^G12D^ mutation, as well as tumor suppressor genes deletion, such as phosphatase and tensin homolog (PTEN) on chromosome 10. Thus, targeting tumor energy metabolism by studying key rate‐limiting enzymes in the PPP process might be a promising cancer therapeutic strategy [31].

G6PDH is the first rate‐limiting enzyme in the oxidative stage of the PPP, which dehydrogenates G‐6‐P to NADPH and 6‐Phosphogluconolactone. However, overactivating the cancer‐promoting signaling pathways, such as PI3K/AKT, Ras, and Src, may promote G6PDH activation through posttranslational mechanisms [30]. Oncogene T‐cell leukemia 1(Tcl1) is an Akt co‐activator that promotes G6PDH activity, while PTEN inactivates Tcl1 through glycogen synthase 3β‐mediated phosphorylation [32]. Studies on the mechanism of HNSC progression showed that c‐Myc induces extensive metabolic reprogramming by depending on nuclear factor erythroid 2‐related factor 2 (NRF2), and G6PDH and transketolase (TKT) in the PPP are critical downstream targets [33]. Furthermore, disturbed nucleotide biosynthesis resulting from c‐Myc/NRF2/G6PDH‐mediated metabolic reprogramming of PPP promotes the malignant progression of HNSC [33].

6PGL is the second rate‐limiting enzyme in PPP that hydrolyzes 6‐phosphogluconolactone to 6‐phosphogluconic acid. It plays a critical part in cell migration and invasion in HCC and is a potential therapeutic target for its treatment [34]. The third rate‐limiting enzyme of the PPP is 6PGDH, which oxidatively decarboxylates 6‐phosphogluconate to yield NADPH and ribulose 5‐phosphate. In lung cancer cells, P53 accumulation is a major feature resulting from the gene silencing of G6PDH. This is accompanied by increased oxygen consumption, leading to ROS accumulation and damage to tumor cells. However, the rate‐limiting enzymes, 6PGL and 6PGDH, have recently not been sufficiently studied in recent years and require further exploration.

### 2.3 Regulation Molecules in Tumor Glucose Metabolism

Numerous studies have shown that tumor cells majorly regulate key rate‐limiting enzymes or biomacromolecules in metabolic processes using various microRNAs, transcription factors, oncogenes, and tumor suppressor genes to achieve metabolic reprogramming [35].

Tumor tissues are mostly hypoxic because of their rapid proliferation characteristics. HIF‐1 is considered a major transcription factor, to affect adaptive responses of hypoxia and can induce angiogenesis‐ and energy metabolism reprogramming‐associated genes, even under normoxia [36, 37]. The abnormal activation of the PI3K/AKT/protein kinase C/histone deacetylase signaling pathway increases HIF1A gene transcription and HIF‐1 activity [37]. Additionally, HIF‐1 positively regulates many key proteins involved in glucose metabolism, including GLUT1, GLUT3, HK1, HK2, GAPDH, phosphoglycerate kinase 1, PKM2, lactate dehydrogenase A (LDHA), and pyruvate dehydrogenase kinase 1. However, HIF‐1 blocks the mitochondrial TCA cycle and OXPHOS to further enhance aerobic glycolysis‐dependence in tumor cells [38, 39].

Myc signaling is considered the most important oncogenic pathway in human cancer that is frequently amplified. Myc encodes a transcription factor, c‐myc, which regulates many cellular processes, including the disturbance of the TME and evasion of host immune responses [40, 41]. C‐myc regulates genes involved in ribosome and mitochondrial biogenesis, glucose metabolism, and glutamine metabolism [10, 41, 42, 43]. Interestingly, there was minimal overlap observed among the Myc‐signatured metabolic pathways mentioned above, indicating that c‐myc exerts regulatory control over these metabolic processes not through a singular major signaling pathway, but rather by functioning as a comprehensive upstream switch for multiple signaling pathways, which suggests c‐myc might be a promising cancer therapeutic target for cancer treatment.

The PI3K/AKT signaling pathway is a pivotal axis for multiple upstream signaling in tumor cells, aggregating and regulating many downstream transcriptional targets, including mTOR (regulates HIF‐1α and c‐Myc on downstream), Forkhead box protein O1 (FoxO1), GSK3, and other key molecules [44, 45]. mTOR signaling induces the expression of glucose metabolism effectors, including GLUT1, HK2, PGI, PFK, PKM2, and LDHA [44, 46]. FoxO1 negatively regulates glucose and lipid metabolism by inhibiting and reducing glycolytic enzyme and triacylglycerol syntheses, respectively. Akt promotes glucose metabolism in tumor cells by ubiquitinating and degrading FoxO1 through protease phosphorylation. Moreover, substantial evidence suggests that some miRNAs, circular RNAs, and lncRNAs are highly correlated with the expression of many key enzymes involved in glucose metabolism by regulating the PI3K/AKT/mTOR signaling pathway [47, 48, 49, 50, 51].

PTEN is an important tumor suppressor that blocks the activation of downstream pathways by dephosphorylating phosphatidylinositol 3,4,5‐trisphosphate to phosphatidylinositol 4,5‐bisphosphate, which is the signaling molecule in PI3K/AKT, to regulate energy metabolism. It has many downstream pathways, such as GSK3/Wnt/β‐catenin and Ras/extracellular signal‐regulated kinase (ERK)/MAPK pathways [45, 52, 53]. PTEN deletion modulated insulin‐related gluconeogenesis inhibition by blocking FoxO1 and peroxisome proliferator‐activated receptor‐γ (PPARγ) co‐activator 1α (PGC1α) activity. Sterol regulatory‐element binding proteins, which regulate several enzymes in the lipid synthesis pathway, are indirectly regulated by PTEN via the PI3K/AKT signaling pathway. Early growth response 1, p53, and PPARγ activate PTEN transcription by binding to the PTEN promoter [54]. Furthermore, PTEN deletion maintains the sensitivity of tumor cells to chemotherapy by negatively regulating the PI3K‐AKT‐MDM2 pathway to promote the stability and transcriptional activity of p53 [53, 55].

P53 is a transcription factor that mediates multiple cellular responses. It exerts an indirect inhibitory effect on glycolysis by inhibiting nuclear factor kappa B (NF‐κB) and Akt/mTOR in a complex network of signaling pathways. Moreover, p53 has several negative regulatory target enzymes in the process of glycolysis and PPP, such as GLUT1, GLUT4, phosphoglycerate mutase (PGM), pyruvate dehydrogenase kinase 2 (PDK2), and G6PDH [56, 57]. As previously mentioned, p53 is positively and indirectly regulated by PTEN. P53 binds to the PTEN promoter, ensuring it receives excessive stimulation signals to positively regulate it is the transcriptional activity of PTEN's transcriptional activity [54, 58]. Furthermore, it can be mutated by missense mutations in its DNA‐binding domain, losing its original function and gaining new abilities to promote cancer development, such as cell proliferation, angiogenesis, metastasis, and chemoresistance [57].

## 3 Glutamine Metabolism Reprogramming in Tumor Cells

Glucose and glutamine are the primary molecules that undergo significant catabolism in most mammalian cells. Because of the Warburg effect in tumor cells, they prefer to convert glucose into lactate rather than acetyl coenzyme A (acetyl‐CoA); therefore, they depend on glutamine to preserve the progression of TCA cycle through a replenishment reaction to meet the rapid growth rate. This means that glucose and glutamine provide most of the carbon, nitrogen, and acetyl‐CoA reducing equivalents needed to support cancer cell growth and division to participate in several pathways involved in energy production, macromolecule synthesis, ferroptosis, ROS balancing, and signal transmission [6, 59, 60]. Studies have demonstrated that oncogenes and tumor suppressors affect tumor cell growth by regulating glutamine metabolic reprogramming. For example, some oncogenes are activated when glutamine is deficient, which consequently cross‐talk with other energy metabolic pathways.

**FIGURE 2:**
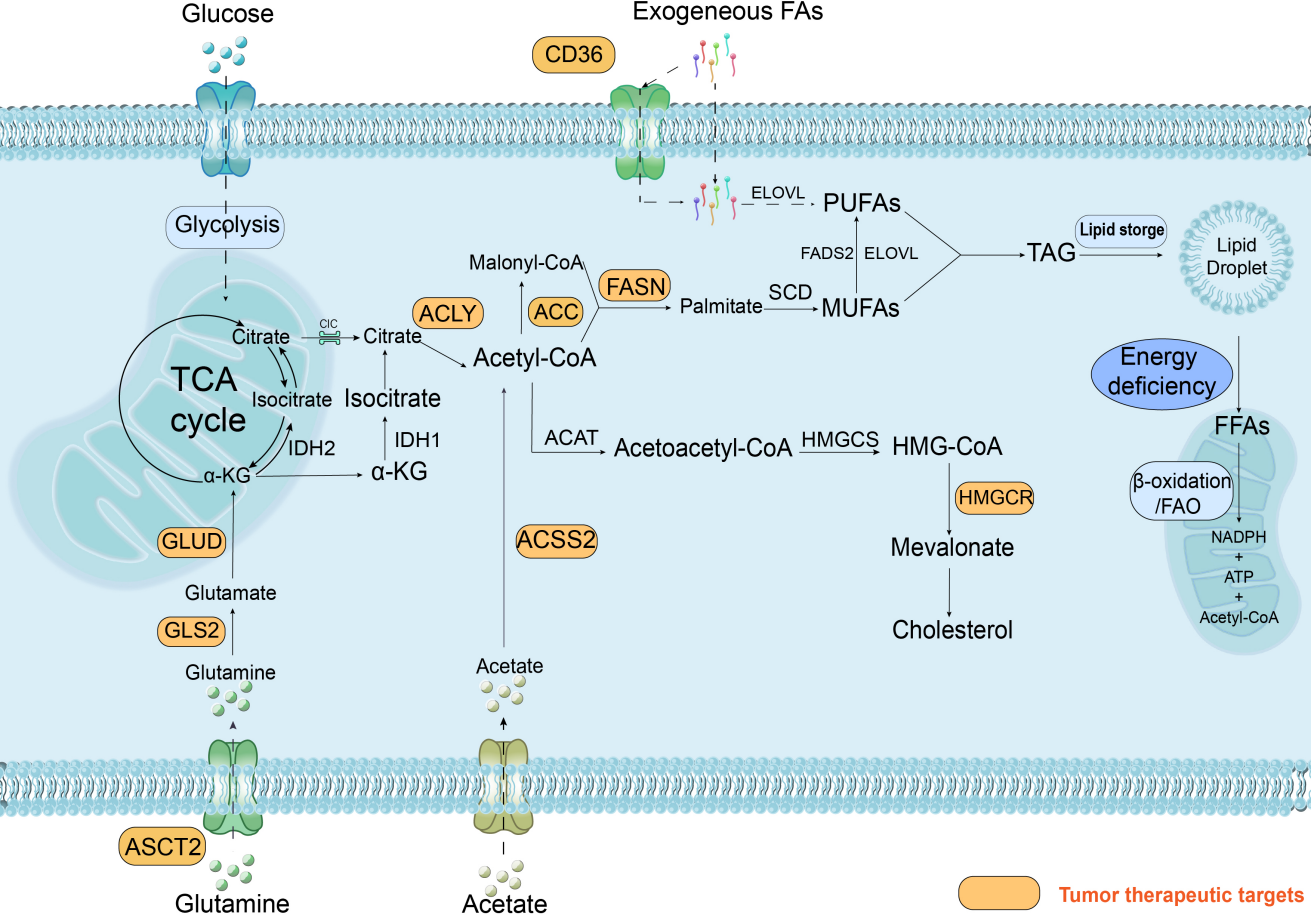
Lipid metabolic pathway. Exogenous fatty acids can be brought into cell via passive diffusion or FA transport or translocase proteins (CD36) across the membrane and be synthesized into PUFAs by the action of elongation of very long lipids protein. As a central substance in lipid metabolism, Acetyl‐CoA can be synthesized from a variety of sources and enter the lipid metabolism process: (1) Exogenous Acetate is synthesized into Acetyl‐CoA by ACSS2 after entering the cell. (2) Citric acid generated from glucose through the TCA cycle is synthesized into Acetyl‐CoA by the action of ACLY. (3) Glutamine is another potential source of acetyl coenzyme A, which is ultimately generated in the cytoplasm through the action of IDH2 and IDH1 in the mitochondria and cytoplasm, respectively. Acetyl coenzyme A can be synthesized into cholesterol by the action of enzymes such as ACAT, HMGCS, and HMGCR. It can also produce FA through ACC, FASN, SCD, and can be stored in the body as lipid droplets after glycerol‐3phosphate as a backbone, and release free fatty acids to generate energy for cells through mitochondrial FAO in energy deficiency. The regulation of key molecules in lipid metabolism is described in detail in the text. α‐KG, α‐ketoglutarate; ACC, acetyl‐CoA carboxylase; ACLY, ATP‐citrate lyase; ACSS2, acetyl‐CoA synthetase 2; ASCT2, alanine, serine, cysteine transporter 2; CD36, cluster of differentiation 36; CIC, citrate carrier; ELOVL, elongation of very long lipids protein; FADS2, fatty acid desaturase 2; FAO, fatty acid oxidation; FASN, fatty acid synthase; FFAs, free fatty acids; GLS2, glutaminase 2; GLUD, glutamine dehydrogenase; HMG‐CoA,3‐Hydroxy‐3‐MethylGlutaryl‐COenzyme A; HMGCR, HMG‐CoA reductase; HMGCS, HMG‐CoA synthetase; IDH1/2, isocitrate dehydrogenase; MUFAs, monounsaturated fatty acids; PUFAs, polyunsaturated fatty acids; SCD, stearoyl‐CoA desaturase; TAG, triacyl glycerol.

The susceptibility genes for aberrant glutamine metabolism in tumor energy metabolism, as well as potential therapeutic targets, are illustrated in Figure 2. Glutamine is initially transported into the cytosol through the amino transporters ASCT2 and SN2, which is a process regulated by the oncogene c‐Myc that binds to the promoter regions of ASCT2 and SN2, upregulating their transcription. ASCT2 is upregulated in several cancers, such as prostate cancer, lung cancer, rectal adenocarcinoma, pancreatic cancer, melanoma, and oral squamous cell carcinoma (OSCC), and has emerged as a potential cancer therapeutic target [61, 62, 63, 64, 65]. Gankyrin, also known as proteasome 26S subunit, non‐ATPase 10 or p28GANK, is a small protein that enhances c‐Myc expression and subsequently upregulates downstream ASCT2 to facilitate glutaminolysis [66]. However, deleting the tumor suppressor liver kinase B1 can increase ASCT2 expression by upregulating HIF‐1, an mTOR‐regulated process that promotes glutamine uptake [67]. Moreover, retinoblastoma protein regulates the downstream transcription factor E2F transcription factor 3, which binds to the ASCT2 promoter to alter glutamine uptake by directly regulating ASCT2 mRNA and its protein expression [68].

The essential rate‐limiting enzyme in glutamine catabolism, Glutaminase (GLS), is depicted in Figure 2, responsible for the conversion of glutamine to glutamate. GLS is highly expressed in various tumor cells, including ovarian cancer and OSCC [65, 69]. GLS exists as two isozymes in mammals contributing distinctively to tumorigenesis as follows: renal glutaminase (GLS1) and hepatic glutaminase (GLS2). GLS1 is positively regulated by c‐Myc and correlates with tumor growth and malignancy, whereas GLS2 is positively regulated by p53 and inhibits the PI3K/AKT pathway to hinder tumor growth [70]. The tumor‐suppressive effect of GLS2 is mainly mediated by the regulation of glutamine catabolism and promotion of ferroptosis and is positively regulated by the tumor suppressor p53 [57, 60]. However, breast cancer studies have shown that GLS2 has carcinogenic effects [71].

α‐ketoglutarate (α‐KG) is generated through two distinct pathways following the conversion of glutamine to glutamate. The first pathway involves using glutamate dehydrogenase (GLUD) to replenish α‐KG in the TCA cycle. GLUD reportedly is expressed in low levels in highly proliferative breast cancer [72]; the second one involves the promotion of other nonessential amino acids production, such as aspartate, alanine, and phosphoserine, through the activity of multiple transaminases, including glutamate‐oxaloacetate transaminase, glutamate‐pyruvate transaminase, and phosphoserine transaminase [73]. Tumor cells with overactivated PI3K, Akt, mTOR, KRas, and c‐Myc positively regulate glutamate metabolism to produce α‐KG, which enters the TCA cycle and provides energy through GLUD or transaminase catalysis [74]. Under hypoglycemic conditions, GLUD plays a crucial role in promoting tumor growth by catalytically generating α‐KG that directly induces the Inhibitor of nuclear factor kappa‐B kinase subunit beta (IKKβ) and NF‐κB signaling, upregulates GLUT1 expression to alleviate glucose deficiency, and facilitates tumor cell proliferation.

## 4 Lipid Metabolism Aberration in Tumor Cells

Lipid metabolism is a pivotal component of metabolic reprogramming in tumor cells in addition to glucose and glutamine metabolism. Many studies have focused on the impact of lipids in the TME on cancer progression. Lipid metabolism changes drastically during cell transformation into a malignant phenotype and is accompanied by enhanced lipid uptake, synthesis, storage, and hydrolysis to supply substances and energy requirements in the subsequent step [75]. Lipid synthesis and its intermediates are essential for cell membrane formation, which is related to substance exchange, signal transduction, and energy generation, particularly in rapidly proliferating cancer cells [75, 76]. Elevated lipogenesis includes *de novo* fatty acid synthesis and cholesterol synthesis. Long‐chain fatty acids (FAs), which are important lipid components, are partially esterified with glycerol to generate triglycerides (TAGs) synthesized and stored in lipid droplets (LDs) under high‐energy conditions. In contrast, TAGs break down to release FAs under low‐energy conditions and undergo FA oxidation (FAO), which is also known as β‐oxidation, in the mitochondria to compensate for the energy deficit [76, 77]. Lipids also function in signal transduction through phospholipase‐dependent hydrolysis of membrane lipids to generate secondary messengers, such as diglycerides, arachidonic acid, and lysophosphate to mediate classical oncogenic signaling pathways, such as Ras and PI3K, further promoting tumor progression [78]. Broadfield et al. reported that ferroptosis, which is a recently identified form of non‐apoptotic polyunsaturated fatty acid (PUFA)‐dependent cell death, is closely associated with regulating lipid metabolism. Thus, tumor cells with high membrane PUFA levels are sensitive to lipid peroxidation and ferroptosis [75]. Moreover, investigating the molecular mechanisms involved in the various stages of lipid metabolism in tumor cells will establish a vital foundation for targeted cancer therapy.

### 4.1 Aberrant Lipid Metabolism Resulting in Increased Lipid Levels

#### 4.1.1 Lipid Uptake

FAs are the precursors of intracellular lipids that perform various functions and regulate gene expression through transcription factors. The uptake of FAs across membrane structure through passive diffusion or transport is the first step in regulating lipid metabolism in cells. FA transporters in the plasma membrane exert many different effects showing different levels of overexpression in tumors, particularly cluster of differentiation 36 (CD36) [79]. CD36 is a multifunctional scavenger receptor that is expressed on the surface of various cell types. The human CD36 gene is located on chromosome 7q11.2, which is associated with the glycoprotein gene family [79, 80]. Additionally, the overexpression of CD36 has been observed in breast, ovarian, gastric, and pancreatic cancers, indicating poor prognosis [81]. Studies have shown that a high‐fat diet upregulates CD36 expression and increases the uptake of FAs by inducing structural changes in CD36 (O‐acylation of S468 and T470 and reduction formation of the C333C272 disulfide bond in CD36), leading to advanced metastasis of gastric cancer [82, 83]. Hypoxia also induces gastric cancer cells to express CD36, enabling the uptake of free fatty acids, consequently leading to peritoneal metastasis [84]. Tumor cells alter the TME for survival and development through autocrine and paracrine secretions, which are characterized by lipid and oxidized lipid enrichment. CD8^+^ tumor‐infiltrating lymphocytes upregulate CD36 expression to promote oxidized low‐density lipoprotein uptake and reduce downstream T‐cell function due to increased peroxide levels in tumor cells [85].

#### 4.1.2 Lipid Synthesis

Although cancer cells consume large quantities of lipids through the exogenous pathway, *de novo* synthesis of their lipids is essential to ensure high‐energy consumption [86]. Glucose and glutamine enter the cell through transporters and participate in the TCA cycle to generate citric acid (carbon transfer from the reduced hydroxylation of glucose and glutamine to citric acid), which is exported across the inner mitochondrial membrane to the cytoplasm via the transporter protein citrate carrier. Citric acid is catalyzed by ATP‐citrate lyase (ACLY) to form acetyl‐CoA, which can be directly catalyzed by acetyl coenzyme A synthase (ACSS) to form acetyl‐CoA in the cytosol via the lactate transporter (monocarboxylate transporters) transporting. Acetyl‐CoA is an important substrate for *de novo* lipid synthesis, generating FAs using enzymes, including acetyl‐CoA carboxylase (ACC), fatty acid synthase (FASN), and stearoyl‐CoA desaturase (SCD). Cholesterol is synthesized from acetyl‐CoA by key enzymes, such as 3‐hydroxy‐3‐methylglutaryl‐CoA reductase (HMGCR) and squalene monooxygenase (Figure 2). Metabolic reprogramming is usually a cascade achieved via changes in related rate‐limiting enzymes, and the overexpression of many lipid synthesis‐associated enzymes is a common feature of many tumor cell types [75].

ACLY is the first rate‐limiting enzyme in *de novo* lipid synthesis that links glucose and lipid metabolism by catalyzing the conversion of citric acid to acetyl‐CoA. Simultaneously, high concentrations of citric acid can inhibit ACLY activity by regulating isomerism [87, 88]. ACLY overexpression has been observed in lung cancer, pancreatic cancer, hepatocellular carcinoma, breast cancer, colorectal cancer, endometrial cancer, and glioblastoma, whereas tumor cells exhibited reduced metastatic activity by reducing low‐density lipoprotein cholesterol levels with phenylpropanoic acid, an ACLY inhibitor [89, 90, 91]. Recent studies revealed that ACLY generates tumor‐promoting effects through many new mechanisms. First, ACLY is required to increase histone acetylation for corresponding growth factor stimulation. Deacetylase SIRT6 regulates cell growth and senescence and is also associated with ACLY. Conversely, mammalian SIRT6 has ACLY‐dependent histone deacetylation activity and can promote the expression of tumor suppressor and aging‐related genes to suppress the aggressive cancer cell phenotypes [92]. Second, ACLY binds to the CTNNB1 gene, which encodes β‐catenin 1 protein, to form and promote complex transport from the cytosol into the nucleus, further increasing the CTNNB1 gene's transcriptional activity and resulting in the migration and invasion of colon cancer [93]. Third, obesity‐related factors, such as insulin and leptin, induce AKT‐mediated phosphorylation of ACLY at Ser455 to promote the nuclear translocation of ACLY, which increases histone acetylation levels and upregulates the pyrimidine metabolism gene DHODH and ultimately promotes tumors [91]. Fourth, IKKβ‐USP30 (ubiquitin‐specific peptidase 30)‐ACLY axis, which is a novel tumor cell regulatory axis, was recently identified to be upregulated in HCC [94]. IKKβ phosphorylates and stabilizes USP30 to deubiquitinate and stabilize ACLY and FASN. Additionally, IKKβ directly acts on ACLY to promote USP30 interaction with ACLY and ACLY deubiquitylation [94]. Lipid synthesis, inflammation, and hepatocarcinogenesis are significantly inhibited without USP30. ACLY, as the first regulatory enzyme for *de novo* lipid synthesis, plays an essential role in reprogramming tumor lipid metabolism.

ACSS2 expression mediates FA synthesis by controlling exogenous acetate utilization. ACSS exists in three isoforms in mammalian cells. ACSS1 and ACSS3 are mitochondrial proteins, and ACSS2 is localized in the cytoplasm and nucleus. As a potential target for cancer therapy, ACSS2 is tightly associated to the cancer stage and patients' survival rate [95]. Additionally, it is highly expressed in glioblastoma, breast cancer, liver cancer, prostate cancer, bladder cancer, renal cancer, and other tumors [95]. Nuclear localized ACSS2 can produce acetyl‐CoA for histone H3 acetylation, leading to brain tumorigenesis [96, 97]. Moreover, the nuclear localization of ACSS2 can be increased by both ischemic and hypoxic glucose deficiency and regulated by the SREBP gene [98]. Consequently, these findings suggest that ACSS2 can be a prospective new target for tumors with highly expressed ACSS2 [95].

ACC is a rate‐limiting enzyme in FA synthesis that catalyzes the carboxylation of acetyl‐CoA to malonyl‐CoA. Two tissue‐specific isoforms, which are ACC1 [encoded by acetyl‐CoA carboxylase alpha (ACACA)] and ACC2 [encoded by acetyl‐CoA carboxylase beta] regulate FA synthesis and oxidation, respectively, exist in mammals [99]. ACC1 expression levels are higher in patients with advanced prostate cancer than in those with early mild disease, suggesting its potential as an early biomarker for prostate cancer [100]. ACC2, which attaches to the mitochondrial membrane, inhibits the mitochondrial uptake process of FA and FAO. It produces malonyl‐CoA that inhibits the activity of carnitine palmitoyltransferase I (also known as CAT1/CCAT), which is an enzyme involved in the rate‐limiting step of FA uptake and FAO [99, 101]. ACC1 is regulated at the transcriptional level by SREBP and by complex interactions involving phosphorylation, allosteric regulator binding, and protein–protein interactions. Preceding studies have shown that the ubiquitin‐specific protease 22 (USP22) promotes FAO by stabilizing sirtuin 1 in the liver [102]. However, a recent study showed that USP22 promotes *de novo* FA synthesis in HCC by promoting ACC and ACLY rather than FAO [103]. USP22 stabilizes PPARγ to increase ACC and ACLY expression, which leads to abnormal lipid metabolism, promoting lipid accumulation and tumorigenesis in HCC cells (this process is accompanied by Akt activation). Furthermore, the abovementioned study confirmed a previously undescribed PPARγ interaction with the peroxisome proliferator response element motif of the ACACA promoter and explained the mechanism of the USP22‐PPARγ/ACC/ACLY axis on HCC development and prognosis [103].

FASN carries malonyl‐CoA and acetyl‐CoA that successively condense into palmitic acid. It is overexpressed in various cancer types, including triple‐negative breast cancer, Wilms tumor, and OSCC [104, 105, 106]. FASN provides a competitive advantage for tumor cell growth and reproduction and is preferentially positively regulated by SREBP1 at the transcriptional level and indirectly regulated passively by p53 due to its inhibition of SREBP1 [57]. For example, mitochondrial elongation factor 2 (MIEF2) upregulates its downstream target genes, ACC1, FASN, and SCD1, by promoting SREBP1 [107]. USP30 directly interacts with FASN leading to its stabilization [94]. Additionally, the stability of FASN at the protein level in breast cancer tissues is regulated by small ubiquitin‐like modifier (SUMO)‐mediated SUMOization of ubiquitin‐like protein modifier molecules, which stabilize FASN from degradation in the protease environment and promote FASN expression [108]. The inhibition of FASN plays an important role in the induction of apoptosis and activates redox‐sensitive kinases that affect mitochondrial apoptotic thresholds, leading to enhanced initiation of mitochondrial apoptosis and apoptotic cell death [109]. Additionally, FASN knockdown would inhibit the proliferation, migration, and invasion of cholangiocarcinoma (CCA) cells, which are KKU055 and KKU213, and induced cell cycle arrest and apoptosis in CCA cell lines [110]. High FASN expression is tightly associated with advanced disease and leads to shortened survival rates in patients with CCA. Recently, FASN has been explored as an effective therapeutic target in tumorigenesis. Furthermore, a selective small‐molecule inhibitor in combination with other drugs can provide considerable synergistic effects in some oncogene‐driven HCC models by inhibiting FASN [111].

HMGCR is a critical rate‐limiting enzyme in the mevalonate pathway of cholesterol production, which converts HMG‐CoA into mevalonate. It is reportedly overexpressed in prostate cancer and is associated with poor prognosis [112]. Myc is upstream of the mevalonate pathway, and the promoter regions of six key enzymes (HMGCR, PMVK, MVK, MVD, IDI1, and FDPS) have Myc‐binding sites; therefore, Myc plays a direct positive regulatory role. Mevalonate also induces miR‐33b to activate Myc signaling [113]. MIEF2 promotes cholesterol biosynthesis by activating ROS/Akt/mTOR signaling to upregulate SREBP2 expression, which is a key transcriptional regulator of cholesterol synthesis, and its transcriptional target cholesterol biosynthesis genes HMGCS1 and HMGCR [107]. HMGCR can be regulated to some extent by changes in the levels of other enzymes during lipid metabolism. For example, the deletion of FASN leads to the compensatory upregulation of HMGCR expression [114]. Therefore, HMGCR is currently recognized as a target for cancer therapy [115].

Reprogrammed FA metabolism, mainly characterized by increased *de novo* lipogenesis, has been progressively established as a cancer symbol. Many key cellular pathways also require increasing lipid levels to facilitate tumor adaptation to nutrient‐poor microenvironments. Thus, studying related enzymes will facilitate the development of targeted interventions for abnormal lipid changes, eliminating cancer at a significantly lower physical cost.

## 5 Metabolic Abnormalities in Tumor Mitochondria

The investigation of the association between mitochondrial metabolic abnormalities and tumorigenesis has emerged as a novel research avenue, garnering significant attention in recent years. Typically, mitochondria are the energy factory of cells that efficiently generate ATP through OXPHOS. Recently, many evidence has recently demonstrated that mitochondria also play important roles in regulating cellular signaling processes, such as scaffolds for protein interaction and regulating levels of intracellular messengers Ca^2+^ or ROS [116, 117, 118]. Mitochondria‐induced metabolic and energy alterations promote tumor progression. For example, increased glycolytic enzyme activity and decreased mitochondrial transcription have been observed at different stages of prostate cancer progression [119]. Transcriptional analysis of 21 tumors in The Cancer Genome Atlas revealed a strong correlation between the inhibition of genes involved in mitochondrial metabolism and unfavorable clinical prognosis, tumorigenesis, invasion, and metastasis [120]. Additionally, the enrichment of mitochondrial DNA (mtDNA) is intricately linked to heightened tumor heterogeneity, while reduced mtDNA levels generally signify impairments in bioenergetics [119]. These suggest that mitochondrial dysfunction plays a pivotal role in driving the malignant progression of tumors by enhancing tumor cell activity, invasiveness, and predisposition to metastasis. Consequently, targeting mitochondrial dysfunction has emerged as a promising therapeutic strategy for combating cancer. The key molecules and regulatory pathways underlying tumor mitochondrial abnormalities have been identified and systematically documented here.

### 5.1 Key Enzymes in Tumor Mitochondria

Mitochondria play an important role in metabolic, energetic, and physiological processes, and their functional integrity is also a critical “checkpoint” for tumor cells. Genetic alterations in some mitochondrial metabolic enzymes, such as fumarate hydratase (FH), succinate dehydrogenase (SDH), and isocitrate dehydrogenase (IDH), result in the accumulation of fumarate, succinate, and 2‐hydroxyglutarate, which promote tumorigenesis and have become promising targets for tumor therapy [121].

### 5.2 Key Molecules Involved in the TCA Cycle in Tumor Mitochondria

The TCA cycle can generate energy by breaking cytoplasmic pyruvate into mitochondria as well as provide reductive NADH and FADH2, which are used for ATP generation via the OXPHOS pathway [122, 123]. As an important metabolic pathway connecting the interconversion of matter and energy, key molecules in the TCA cycle have become the focus of research on the mechanism of tumorigenesis. Recent studies have demonstrated that mutations in certain active mitochondrial enzymes can result in the dysfunction and disruption of the TCA cycle, thereby promoting the development of specific types of cancer. These mutations predominantly occur within several key proteins, including IDH, SDH, and FH. The reduction in TCA cycle flux leads to decreased ATP production through mitochondrial respiration, which may be associated with abnormal energy metabolism affecting tumor formation [122, 123]. The research on these key molecules in recent years is summarized below.

Frequent gene mutations of IDH are observed in various cancer types [124, 125]. The tumorigenic mechanism induced by IDH mutation is closely associated with its intrinsic functionality. IDH facilitates the reversible oxidative decarboxylation of isocitrate to α‐KG. The diminished levels of α‐KG resulting from IDH mutations impact a diverse array of downstream molecules, thereby converting normal cells into tumor cells. The heterozygous mutations of IDH result in a depletion of α‐KG production, which can be taken up by CD8+ T‐cells to generate and accumulate the cancer metabolite D‐hydroxyglutarate. This leads to an acute and reversible impairment of its metabolism and antitumor function, ultimately contributing to the development of tumors commonly observed in glioblastoma [126]. α‐KG is also an important component in HIF degradation, and a decrease in its concentration upregulates the level of HIF‐1α, which is a significant factor contributing to tumor progression in heterozygous mutants of IDH and why IDH plays a crucial role in tumor suppression by catalyzing α‐KG production [127]. Moreover, tumor suppression by p53 partly depends on α‐KG and antagonizes malignant progression by promoting tumor cell differentiation [128]. The isoenzyme IDH1, a member of the IDH family, exhibits significant upregulation and enhances cell migration in patients with primary gliomas. Wild‐type IDH1 regulates the migration of primary glioma cells by catalyzing the production of α‐KG, which mediates the activation of the PI3K/AKT/mTOR pathway cascade phosphorylation. The PI3K/AKT/mTORC1‐HIF1α axis additionally promotes glucose metabolic reprogramming, leading to the overexpression of GLUT1, thereby suggesting that both GLUT1 and mutant IDH1 could serve as potential targets for various cancers, including colorectal cancer and glioblastoma [129, 130, 131].

SDH is a mitochondrial enzyme, which generates fumarate using succinate as a substrate, and is a hub that connects OXPHOS and electron transport chains (ETCs). The SDH mutation leads to dysfunction of complex II and enables tumor cells to adapt to the complex environment by altering downstream metabolism. For instance, inhibition of prolyl hydroxylase (PHD), resulting in increased activity of HIFα, can induce persistent HIFα expression even under normal oxygen conditions, facilitating tumor cell adaptation to hypoxic environments [132]. Myc triggers tumor‐specific gene expression by promoting the acetylation‐dependent inactivation of SDHA (one of an important isoenzymes of SDH), inhibiting SDH activity, leading to succinate accumulation, and accelerating tumorigenesis both in vitro and in vivo [133]. SDH mutation or inactivation is indispensable to the role of many cancer‐promoting star molecules, which shows the importance of SDH in cancer research.

The FH and fumarate are recognized as tumor suppressors and oncogenic metabolites, respectively. Mutations in FH, similar to SDH mutations, impede PHD activity, thereby stabilizing HIFα to facilitate the adaptation of tumor cells to hypoxic conditions [132, 134]. Specifically, FH deletion suppresses AMPK activation to further cross‐activate mTOR and ACC thus promoting lipid biosynthesis to provide energy material for tumor growth [134]. Notably, the activation of mTORC1 can downregulate the expression level of FH by deleting tuberous sclerosis factor 1/2 (TSC1/2), generating bidirectional regulation between FH and mTOR [134]. The levels of fumarate are elevated in FH‐deficient cells, leading to direct interaction with cysteine 211 of PTEN and subsequent activation of the PI3K/AKT signaling pathway through inhibition of PTEN [135]. Fumarate competitively inhibits the α‐ketoglutarate‐dependent dioxygenase family that encompasses Jumonji C‐domain lysine demethylases (JmjC‐KDMs), ten‐eleven translocation (TET) DNA cytosine‐oxidizing enzymes, and other related enzymes. TET enzymes facilitate the oxidation of methylated cytosines on DNA for efficient demethylation processes. Additionally, JmjC‐KDMs are capable of removing methylation marks from histone tails. Consequently, FH mutations induce cellular DNA hypermethylation through these mechanisms, ultimately contributing to tumorigenesis [136].

### 5.3 Oxidative Phosphorylation in Tumor Mitochondria

**FIGURE 3:**
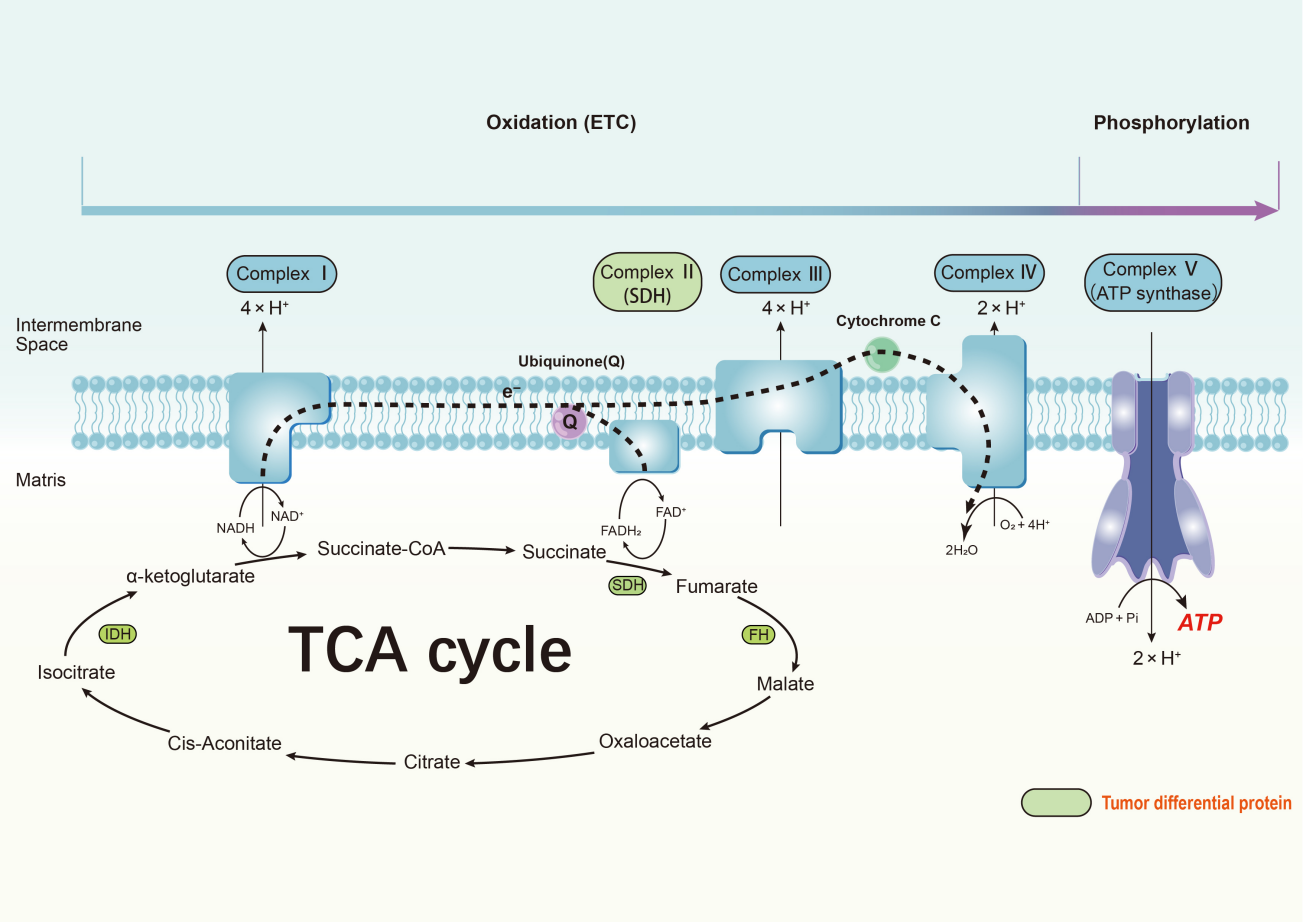
Mitochondrial energy metabolism. Energy metabolic processes occurring in mitochondria mainly include the TCA cycle and oxidative phosphorylation. Oxidative phosphorylation has been classified as (1) oxidizing phase: Transfer of electrons from reducing coenzymes through complexes I–IV to the electron terminal receptor “O_2_” (this process is also known as ETC); (2) TCA cycle: The energy released by ETC is stored in an electrochemical gradient that catalyzes the complex V that phosphorylates ADP to ATP. Complexes I/II are electron‐receiving portals in the oxidative phosphorylation pathway, receiving electrons from NADH and FADH2, respectively, but complex II does not exercise the function of pumping H^+^ to the intermembrane space. Complexes I/III/IV utilize the energy released during the transfer of electrons to pump H^+^ outward. Because complexes I–IV are anchored to the inner mitochondrial membrane, they require the lipid‐soluble electron transport “messengers”, cytochrome c and coenzyme Q (ubiquinone), in order to complete the process of ATP formation. Complex II plays a dual role in cellular energy metabolism because it is also a key enzyme in the TCA cycle. Mutations in IDH, FH, and SDH, genes encoding key enzymes in the TCA cycle, reprogram energy metabolism and promote the development of certain cancers. complexes I, NADH: ubiquinone oxidoreductase; complex II, succinate dehydrogenase; complex III, cytochrome bc1; complex IV, cytochrome c oxidase; complex V, ATP synthase; ETC, electron transport chain; FH, fumarate hydratase; IDH, isocitrate dehydrogenase; SDH, succinate dehydrogenase.

The mitochondrial OXPHOS system is the final biochemical pathway in ATP production that plays a significant role in macromolecular anabolism in cancer cells (Figure 3) and has become an emerging target for tumor therapy [137]. The energy released during glycolysis, the TCA cycle, and β‐oxidation is mostly stored in reductive coenzymes and produces ATP through the OXPHOS pathway. The OXPHOS metabolic pathway transports electrons through several transmembrane protein complexes (I–IV) on the inner mitochondrial membrane. The protons pumped from complexes I, III, and IV into the intermembrane space are accompanied by a high proton gradient through complex V (ATP synthase), driving ATP synthesis in the ETC [138]. Due to the important role of OXPHOS in tumor energy metabolic reprogramming, the following is a detailed review of important complexes, potential therapeutic targets, and tumor characteristic molecules in OXPHOS.

1. Relationship between oxidative phosphorylation and aerobic glycolysis in tumor cells

Contrary to the conventional theory of the relationship between OXPHOS and aerobic glycolysis in tumor cells decades ago (Warburg effect), recent studies have found that some cancer cells use OXPHOS as the primary route of ATP production, and highly metastatic tumor stem cells with oncogenic potential appear more dependent on OXPHOS [139]. For example, reduced levels of glycolysis and dependence on high OXPHOS levels have been demonstrated in studies related to KRas‐mediated pancreatic ductal adenocarcinoma stem cells [140]. OXPHOS has also been shown in Hodgkin lymphoma to be higher in normal cells, with NF‐κB promoting the expression of genes including OXPHOS, mitochondrial genesis biomarker levels, increased ETC key proteins, and decreased lactate production (suggesting reduced glycolysis levels) [141]. High OXPHOS levels are present even when competing glycolytic levels remain active; although this phenomenon is currently targeted only for certain cancer types, this evidence is sufficient for the traditional Warburg effect theory to pose a challenge [138]. Cancer cells can gradually compensate for the dumping from high glycolysis to OXPHOS even in the same tumor to maintain oxidative metabolism, a phenomenon known as cancer cell metabolic plasticity or mixed metabolism, which depends on a highly heterogeneous TME [142]. Various studies have uncovered the potential of OXPHOS as a new target for cancer therapy, and providing novel insights into overcoming the challenge of high tumor tolerance to conventional chemotherapeutic agents [143].

2 Tumorigenesis‐associated OXPHOS complexes

Increased dependence on OXPHOS tends to characterize cancer stem cells as well as cells resistant to chemotherapy and targeted therapies. Inhibition of OXPHOS function may also affect the tumor microenvironment by alleviating hypoxia and improving anti‐tumor immune responses. Therefore, targeting OXPHOS is a promising strategy for the treatment of various cancers [143]. Six important enzymes (complexes I–V, and electron transfer flavoprotein: ubiquinone oxidoreductases) located in the inner mitochondrial membrane are involved in OXPHOS. Recent research on each of the complexes is summarized separately below.

NADH: ubiquinone oxidoreductase (complex I) is the first electron entry point in the ETC and the largest complex with 46 subunits. Complex I oxidizes NADH to NAD^+^ in the mitochondrial matrix and reduces ubiquinone to ubiquinol [144]. Multiple subunits of complex I show different expression characteristics in different cancer types; for example, NADH Dehydrogenase [Ubiquinone] 1 Alpha Subcomplex Subunit 4‐Like 2 (NDUFA4L2) is overexpressed in colorectal cancer and clear cell renal cell carcinoma [145]. NDUFA10 and NDUFV2 have also emerged as potential prognostic markers for prostate cancer [145, 146, 147]. Complex I expression is associated strongly with conventional cancer therapies, such as radiotherapy and chemotherapy. Recent research showed that resistance to conventional therapies in patients with tumors is also due to the overexpression of complex I in tumor cells [148, 149]. Besides, Complex I is relevant with fractionated radiation‐induced radioresistance in gliomas and is an effective target for clinical radiation therapy of gliomas. The sensitivity of glioma cells to radiation can be enhanced by inhibition of Complex I. This may be owing to the effect of complex I on ROS production, which interferes with radiolucent efficacy [148].

SDH/Complex II comprises four subunits encoded by nuclear DNA in a head‐to‐tail arrangement as follows: SDHA/B/C/D. SDH is the only complex involved in both the TCA cycle and the ETC. Reduced expression of SDH subunits has been detected in various cancers, including brain cancer, breast cancer, and pediatric acute myeloid leukemia [150, 151, 152]. Loss of SDHB immunohistochemical staining is a hallmark of these tumors [153]. Furthermore, SDHB acts as a tumor suppressor and its inactivation is widely associated with cancer malignancy [154, 155, 156]. Succinate, a substrate for SDH, accumulates and is secreted into the cytoplasm and extracellular environment owing to SDH subunits mutation or reduced expression. Recent reports indicate that cancer‐secreted succinic enhances cancer cell migration and promotes cancer metastasis by activating succinate receptor‐1 (SUCNR‐1)‐mediated signaling and transcriptional pathways [157].

Cytochrome bc1 (complex III) is the third enzyme in the mitochondrial respiratory chain, to which distinct substrate‐dependent branches of the chain converge. It transfers electrons from ubiquinol to cytochrome c and contributes to generating an electrochemical proton gradient [144]. Numerous subunits of complex III are closely associated with tumor development. For example, ubiquinol‐cytochrome C reductase‐binding protein (UQCRB) is overexpressed in human colon carcinoma cells [158]. Notably, a homozygous mutation in UQCRB is associated with the defective function of mitochondrial complex III [159]. Studies suggest that UQCRB overexpression‐induced elevated mitochondrial ROS levels in human colon cancer cells induce autophagy and that UQCRB is a new molecular prognostic biomarker of colorectal cancer [158, 160]. Another subunit, ubiquinol‐cytochrome C reductase core protein II (QCR2), is indispensable for complex III functionality and is upregulated in the lung, liver, breast, thyroid, cervical, esophageal, and prostate tumor specimens [156]. QCR2 mediates the ubiquitination and degradation of p53 by directly interacting with prohibitin (PHB), which is the molecular chaperone of p53 in the mitochondria, to prevent it from binding to p53 in the nucleus [156].

Cytochrome c oxidase (COX/complex IV) comprises 14 subunits in mammals (mtDNA encodes subunits I–III) and has diverse interactions with complex I [143]. Colorectal cancer cells show higher COX expression than ulcerative or normal colon epithelial cells. Particularly, the expression level of subunit IV, which plays an important role in COX assembly, was most significantly increased; however, it was not associated with cell proliferation [161]. COX Va is upregulated in lung cancer cells and renal cell carcinoma [162, 163]. Cytochrome c oxidase assembly protein 2 (SCO2) is essential for complex IV function. However, the loss of function or deletion of SCO2 leads to the abrogation of COX activity and increases in NADH, ROS, and glycolysis, which are accompanied by the reversed activity of ATP synthase [164]. Since SCO2 expression is regulated by p53, metabolic disturbances caused by p53 loss are considered to result, at least in part, from SCO2 downregulation [165].

ATP synthase (complex V) is an endpoint enzyme in OXPHOS comprising 16 subunits. Complex V drives the reversible phosphorylation of ADP and phosphate to produce ATP by returning H^+^ via the proton pump [144]. ATP synthase comprises soluble F1 and membrane‐spanning F0 portions. The catalytic active site is the β‐subunit of the F1 portion, which is encoded by ATP synthase β‐subunit (ATP5B) and has been suggested as a potential gastric cancer marker and a new target for paraganglioma [166, 167, 168, 169]. ATP5B is highly expressed in human epidermal growth factor receptor 2‐positive breast cancer, highly metastatic prostate cancer, gastric cancer, and glioblastoma [168, 169, 170, 171]. Interestingly, studies have revealed the phenomenon that ATP synthase, which is supposed to be localized in the inner mitochondrial membrane, shows translocation of cytosolic expression in some tumor cells. Ectopic expression of the complex V, including ATP5B, on the surface of tumor cell membranes promotes cell proliferation, possibly facilitating the adaptation of cells to the hyper‐acidic environment of glycolysis, and this provides conditions for tumor proliferation and migration [166, 172, 173]. Recent study suggested that, the cancer‐promoting effect of ATP5B is produced by transporting ATP outside the cell membrane to bind the ATP receptor (P2X7) and activate the focal adhesion kinase/AKT/matrix metalloprotease 2 signaling pathway to promote the proliferation and migration of gastric cancer cells [168]. ATP synthase is a potential therapeutic target and plays a crucial role in maintaining reversible drug resistance in certain tumor cell types, such as breast tumors. These resistant strains are more sensitive to OXPHOS inhibitors and can be reversed by applying OXPHOS inhibitors to accomplish more efficient oncological treatments [170]. But previous study noted the prevailing dogma that “malignant cells express more ectopic ATP synthase than less malignant one” is not necessarily true, thus the potential of ectopic expression of the complex V as a therapeutic target for tumors remains to be evaluated [172].

3 Key regulatory molecules of abnormal OXPHOS of tumor cells

The traditional Warburg effect indicates that many cancer cells have downregulated OXPHOS, which may be associated with ROS upregulation owing to mtDNA mutations and reduced expression of key protein subunits of the respiratory chain for DNA translation. Meanwhile, the inhibition of KRas‐induced cancer progression in mice with lung cancer after gene knockdown, and the reduction in respiratory chain complex biosynthesis, demonstrated the absolute requirement of mitochondrial respiratory chain proteins for tumor cell progression [174, 175]. As the most important cancer‐promoting molecule Myc, chromosome immunoprecipitation‐sequencing (ChIP‐seq) analysis yielded over 400 nuclear‐encoded mitochondrial genes, including genes associated with OXPHOS complexes, mitochondrial transcription/translation factors, mitochondrial ribosomes, and other transcription factors involved in mitochondrial biogenesis, which were identified as Myc targets. In prostate cancer, Myc indirectly affects the levels of these proteins and targets the mitochondrial chaperone protein tumor necrosis factor receptor‐associated protein 1, which mediates mitochondrial protein folding and functional levels of OXPHOS [176].

PGC1α is a key transcriptional regulator of mitochondrial OXPHOS that can promote tumor progression and metastasis; its overexpression in prostate cancer cell lines with undetectable PGC1α induces OXPHOS, thereby reversing the Warburg effect; and the oncogenic or tumor suppressor effects of PGC1α, as well as the induction of OXPHOS, are highly situation‐dependent [176]. PGC1α is a hub for multiple signaling pathways in prostate cancer. First, it interacts directly with the androgen receptor (AR) and activates its transcriptional activity; however, related studies have shown that AR signaling positively feeds back through the AMPK pathway to induce PGC1α, a signaling pathway axis that controls the metabolic activity of prostate cancer [177]. Furthermore, PGC1α also affects estrogen‐related receptor (ERRα)‐related signaling; the PGC1α–ERRα axis is repressed in prostate cancer, and this axis has potential tumor‐suppressive effects and downregulates Myc [178].

The expression of the methyltransferase NOP2/Sun RNA methyltransferase 3 (NSUN3) further enhanced the power of mitochondrial OXPHOS by increasing the occurrence of cytosine methylation modification to 5‐methylcytosine on transfer RNA in cancer cell mitochondria, leading to an upregulation of gene expression levels and a consequent significant increase in the protein fraction of the respiratory chain. Therefore, the adequate provision of ATP significantly increased the metastatic invasive potential of tumor cells, and subsequent controlled experiments determined the potential of NSUN3 as a therapeutic target for tumors. Notably, the absence of NSUN3 did not affect primary tumorigenesis but specifically affected the metastatic invasive potential of tumors [179].

## 6 Conclusion and Perspectives

The altered energy metabolism has been firmly established as a significant phenotypic characteristic of tumor cells over the past few decades. Additionally, reprogramming of glucose, FA, amino acid metabolism, and OXPHOS confers an unlimited growth potential to cancer cells. The fundamental concept underlying cancer therapy is to enhance treatment efficacy through a comprehensive understanding of the intricate mechanisms governing energy metabolic pathways and their intricate interplay in the regulation of tumorigenesis. However, targeting altered metabolism can be a viable approach for cancer therapy by developing small‐molecule inhibitors of metabolic enzymes and applying emerging immunotherapies to treat malignant tumors. Therefore, we conducted a comprehensive examination of the molecules that regulate crucial energy metabolic networks in the reviewed studies (Table 1). Researchers have developed novel inhibitors targeting mtDNA that restrict mitochondrial formation through the highly selective inhibition of the catalytic activity of the human RNA polymerase mitochondrial. Compared with previous mitochondrial inhibition techniques, this inhibitor impeded cancer cell proliferation and tumor growth in mice without significantly affecting healthy cells [180]. However, the shortcomings of various studies have gradually been revealed with the progress of research. Tumor cells can steal organelles, including mitochondria, from the surrounding immune cells by forming nanotubes, thereby obtaining a large amount of energy for their growth and increasing chemoresistance [181]. This implies that regarding therapeutic strategies, inhibition of a single enzyme or pathway may be insufficient to realize the full potential of targeted therapies in cancer treatment. Furthermore, considering the complex framework of energy generation over the formation and intermediates, including dynamic interactions with the TME and nutrient availability, is necessary with great promise for implementing new combinatorial strategies. Therefore, this review systematically summarizes the characteristics of energy metabolism and gene expression abnormalities in tumors, providing strong support for tumor therapies that target molecules or pathways, which result in energy metabolism abnormalities.

**TABLE 1:** Key molecules in the process of tumor energy metabolism.

	Key molecules	Function	Expression and Reference
Glucose Metabolism	GLUT	Uptaking extracellular glucose into intracellular	Upregulation in most tumors, such as breast cancer, pancreatic, lung, renal, cutaneous, gastric, and esophageal tumors [9].
	HK	Phosphorylating glucose to G‐6‐P	Upregulation in HNSC, cholangiocarcinoma, lung squamous cell carcinoma, prostate adenocarcinoma, stomach adenocarcinoma, esophageal carcinoma, glioblastoma multiforme, bladder urothelial carcinoma [13].
	PFK‐1	Catalyzing F‐6‐P to F‐1,6‐BP	Upregulation in cervical carcinoma, breast carcinoma [19, 20].
	PK	Catalyzing PEP to produce ATP and Pyruvate	Upregulation in various cancer cell types [24].
Glutamine Metabolism	ASCT2	Uptaking glutamine into the cytosol	Upregulation in prostate cancer, lung cancer, rectal adenocarcinoma, pancreatic cancer, melanoma, and oral squamous cell carcinoma [61, 62, 63, 64, 65].
	GLS	Regulating glutamine catabolism	Upregulation in ovarian cancer and OSCC [65, 69].
	GLUD	Converting glutamine to α‐ketoglutarate	Downregulation in highly proliferative breast cancer [72].
Lipid Metabolism	CD36	Uptaking FA into the cytosol	Upregulation in breast, ovarian, gastric, and pancreatic cancers [81].
	ACLY	Catalyzing citrate to acetyl coenzyme	Upregulation in lung cancer, pancreatic cancer, hepatocellular carcinoma, breast cancer, colorectal cancer, endometrial cancer, and glioblastoma [89, 90, 91].
	ACSS2	Catalyzing the production of acetyl coenzyme from exogenous acetic acid	Upregulation in glioblastoma, breast cancer, liver cancer, prostate cancer, bladder cancer, renal cancer [95].
	ACC	Catalyzing Acetyl‐CoA to Malonyl‐CoA	Upregulation in prostate cancer [100].
	FASN	Condensing Malonyl‐CoA and Acetyl‐CoA successively to produce Palmitate	Upregulation in triple‐negative breast cancer, Wilms tumor, and OSCC, CCA [104, 105, 106, 110].
	HMGCR	Reducing HMG‐CoA to Mevalonate	Upregulation in Pca [112].
Oxidative phosphorylation	Complex I	Transferring electrons to ubiquinol and pumping proton	NDUFA4L2 → upregulation in colorectal cancer, clear cell renal cell carcinoma [145].
	Complex II	Transferring electrons to ubiquinol	SDH → downregulation in brain cancer, breast cancer, pediatric acute myeloid leukemia [150, 151, 152].
	Complex III	Transferring electrons to cytochrome c and pumping proton	QCR2 → upregulation in lung, liver, breast, thyroid, cervical, esophageal, prostate cancer [156].
			UQCRB → upregulation in colon carcinomas [158].
	Complex IV	Transferring electrons to oxygen and pumping protons	COX IV → upregulation in Colorectal cancer cells [161].
			COX Va → upregulation in lung cancer, renal cell carcinoma [162, 163].
	Complex V	Catalyzing ADP phosphorylation to ATP	ATP5B → upregulation in HER2‐positive breast cancer, highly metastatic prostate cancer, gastric cancer, glioblastoma [168, 169, 170, 171].

## Author Contributions

All authors contributed to the conception and the main idea of the work. S.F. and Y.X. developed the idea and drafted the main text. H.X. amended the manuscript. S.F. and J.G. designed the tables and figures. S.F. and H.N. contributed to this work mainly on investigation. H.X. and Y.X. supervised the work and provided the comments and additional scientific information. All authors read and approved the final version of the work to be published.

## Ethics Statement

The authors have nothing to report.

## Consent

All authors have reviewed and approved the publication of this review.

## Conflicts of Interest

The authors declare no conflicts of interest.

## Data Availability

This study did not generate new data. All findings summarized in this manuscript come from articles cited in the reference list.
